# Safety and efficacy of radiotherapy combined with lenvatinib plus PD-1 inhibitors as neo-adjuvant therapy in hepatocellular carcinoma with portal vein thrombus: protocol of an open-label, single-arm, prospective, multi-center phase I trial

**DOI:** 10.3389/fonc.2022.1051916

**Published:** 2022-11-24

**Authors:** Guangxin Li, Bin Shu, Zhuozhao Zheng, Hongfang Yin, Chen Zhang, Ying Xiao, Yanmei Yang, Zhe Yan, Xiaofei Zhang, Shizhong Yang, Gong Li, Jiahong Dong

**Affiliations:** ^1^ Department of Radiation Oncology, Beijing Tsinghua Changgung Hospital, School of Clinical Medicine, Tsinghua University, Beijing, China; ^2^ Hepatopancereatobiliary Center, Beijing Tsinghua Changgung Hospital, School of Clinical Medicine, Tsinghua University, Beijing, China; ^3^ Department of Radiology, Beijing Tsinghua Changgung Hospital, School of Clinical Medicine, Tsinghua University, Beijing, China; ^4^ Department of Pathology, Beijing Tsinghua Changgung Hospital, School of Clinical Medicine, Tsinghua University, Beijing, China; ^5^ Center for Clinical Epidemiology & Biostatistics, Beijing Tsinghua Changgung hospital, School of Clinical Medicine, Tsinghua University, Beijing, China; ^6^ Research Unit of Precision Hepatobiliary Surgery Paradigm, Chinese Academy of Medical Sciences, Beijing, China

**Keywords:** radiotherapy, lenvatinib, sintilimab, HCC, PVTT

## Abstract

**Background:**

Surgical resection is a mainstay to treat hepatocellular carcinoma (HCC) with portal vein tumor thrombus (PVTT) in east Asia. However, the postoperative recurrence rate is high. It is necessary to explore neo-adjuvant therapy to increase the surgical resection rate and improve overall survival. Evidence has shown that lenvatinib combined with PD-1 inhibitors is safe and effective in the treatment of advanced unresectable HCC. Radiotherapy is also an effective treatment method for PVTT and has a synergistic effect in combination with PD-1 inhibitors. Surgical resection after Lenvatinib and sintilimab combined with radiotherapy as a neoadjuvant treatment regimen may be a new exploration of HCC with PVTT, but there were not any reported.

**Methods:**

This open-label, single-arm, prospective, multi-center Phase I trial will enroll 20 HCC patients with PVTT who have a resectable primary tumor and no extra-hepatic metastasis. Eligible patients will be given radiotherapy, 3Gy*10 fraction, and will receive lenvatinib 8-12mg once daily and sintilimab 200mg once every three weeks. Surgical resection will be performed 6-8 weeks after radiotherapy. The primary endpoint is safety (number of patients ≥3G TRAE) and the number of patients who complete pre-op treatment and proceed to surgery. The secondary study endpoints include Major Pathological Response (MPR), 1-year tumor recurrence-free rate, Objective Response Rate (ORR), Imaging-Pathology Concordance Rate (IPCR), PVTT regression rate, Median Overall Survival (OS) and Recurrence Free Survival (RFS).

**Discussion:**

This trial may confirm that surgical resection following intensive neoadjuvant therapy can provide a safe and efficient regimen for BCLC stage C patients with PVTT.

**Clinical trial registration:**

https://clinicaltrials.gov/, identifier (NCT05225116).

## Introduction

Portal vein tumor thrombus (PVTT), having biological behavior of vascular invasion, is common in patients who are first diagnosed with hepatocellular carcinoma (HCC). The incidence of PVTT varies between countries and regions, ranging from 13% to 45% (1). HCC patients with PVTT have a worse prognosis, with a median survival of only 4-6 months given the best supportive care (2, 3). The management guidelines of HCC in the United States and ESMO recommend sorafenib and Lenvatinib as first line systemic therapy for PVTT patients (4, 5). However, the efficacy is modest. Guidelines for the management of HCC in Asia, such as Asian-Pacific guidelines (6) and guidelines in mainland China (7), Korea (8), and Taiwan (9), suggest that local therapies (hepatic resection, radiotherapy, TACE, and HAIC for example) are optional regimen for patients with PVTT. A real-world study in Japan reported postoperative recurrence-free survival according to the degree of PVTT as follows: Vp1, 1.23 years; Vp2, 0.82 years; Vp3, 0.56 years and Vp4, 0.38 years (10). The clinical benefit is unsatisfactory either. Till now, there is no global consensus or standard guidelines for the treatment of HCC patients with PVTT, along with an urgency to find new treatments.

Recently, immunotherapy marks a new dawn in HCC management. IMbrave 150 study demonstrated an improvement in clinical benefit with atezolizumab (anti-PDL1 antibody) and bevacizumab (anti-VEGF antibody). According to IMbrave 150, 129 patients with macrovascular invasion included in the study had mOS of 14.2 months vs. 9.7 months (HR 0.68)) and mPFS of 6.7 months vs. 4.2 months (HR 0.59), which confirmed the effectiveness of ICIs combined with VEGF inhibitor in patients with PVTT. However, in 73 patients with Vp4 PVTT, the OS was 7.6 months, which is still unsatisfactory (11). In a prospective study by Lu et al., examined the efficacy of PD-1 inhibitors combined with Lenvatinib in HCC patients with major vascular invasion as conversion therapy. Successful conversion rate was 42.4%; median overall survival was 6.5 months (12).

Radiotherapy is increasingly used in advanced HCC and demonstrated encouraging clinical benefit in management of PVTT. Cheng et al. found that neoadjuvant radiotherapy reduced the extent of PVTT and improved post-operative survival rate reaching 75.2% at 12 month (13). In addition, radiotherapy upregulated PD-L1 in patients with HCC, potentiated the antitumor effect of immune checkpoint inhibitors and augmented cytotoxic T-cell infiltration in HCC tumors in immunocompetent mice (14).

Thus, we designed this study to evaluate the safety and efficacy of radiotherapy combined with lenvatinib plus PD-1 inhibitors as neo-adjuvant therapy in hepatocellular carcinoma with portal vein thrombus.

## Methods and analysis

### Study design

**Figure 1:**
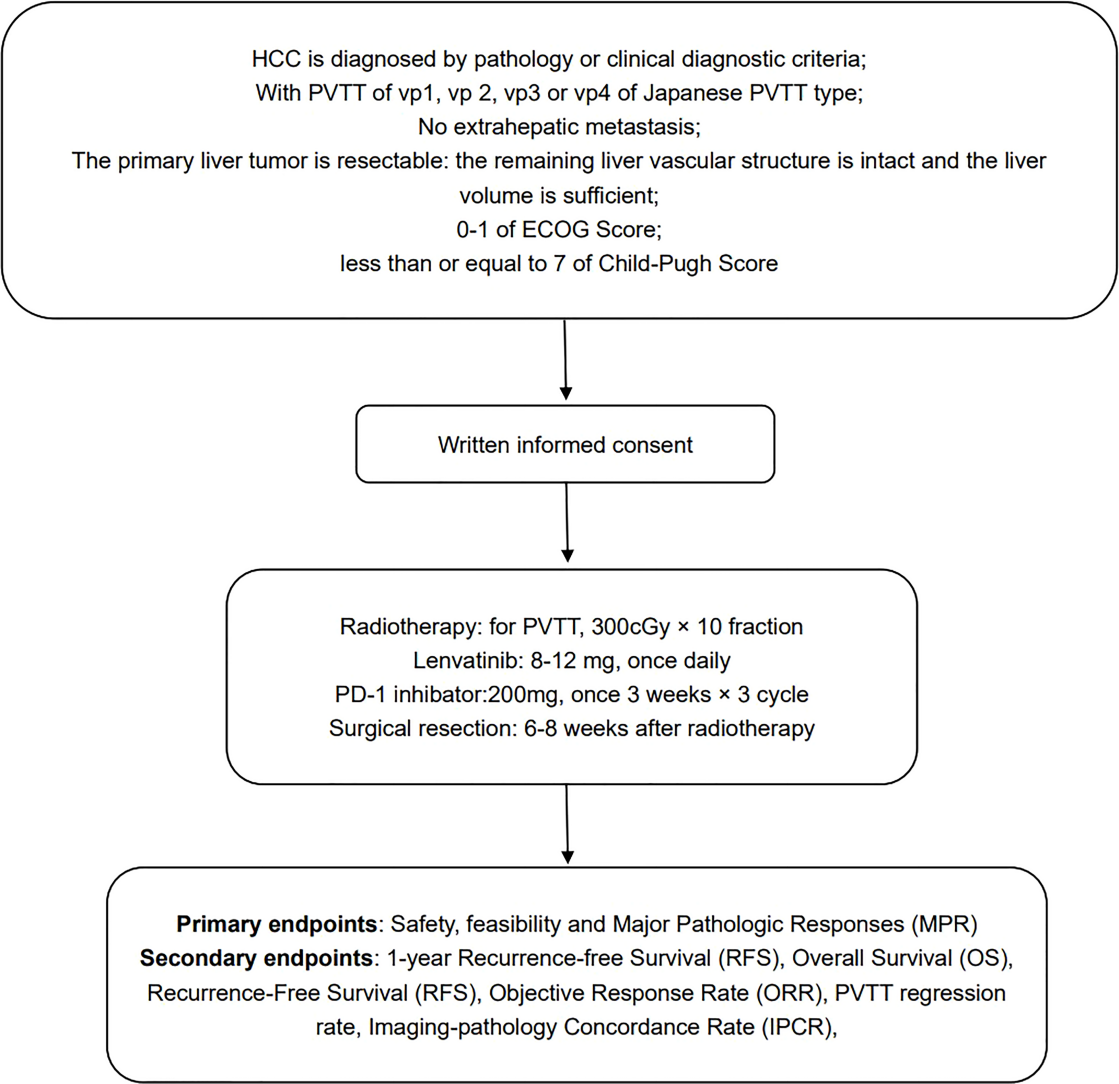
Study design.

This is an open-label, single-arm, prospective, multi-center phase I trial in HCC patients with portal vein thrombus which will be conducted in 5 hospitals in China. (**Figure 1**). The study is being followed the Declaration of Helsinki and Good Clinical Practice. The protocol and its amendments have been approved by the ethics committee of Beijing Tsinghua Changgung Hospital (No. 21323-0-03). The recruitment started on December 01, 2022. The enrolment is estimated to complete in December 1, 2025.

### Selection of subjects

#### Eligibility criteria

The patient inclusion and exclusion criteria are detailed in **Table 1**.

**Table 1:** Eligibility criteria.

Inclusion criteria	
1	Aged 18-70 years, no gender restrictions
2	Been diagnosed with HCC by histopathological or cytological examinations or meet the Chinese clinical diagnostic criteria of the "Guidelines for the Diagnosis and Treatment of Primary Liver Cancer" (2019 Edition)
3	Tumor thrombus in the main portal vein or branches (vp1, vp2, vp3 or vp4 of Japanese PVTT type) and without extrahepatic metastasis
4	The primary tumor is resectable: the remaining liver vascular structure is intact and the liver volume is sufficient, which is in line with the decision-making system for safe hepatectomy
5	ECOG performance status 0-1
6	Child-Pugh score ≤7
7	HBV DNA <500 IU/ml and have been receiving conventional antiviral therapy for HBV antigen-positive patients
8	For normal function of major organs, the following criteria should be met: Adequate bone marrow function, defined as: Absolute neutrophil count (ANC ≥1.5 x 10^9 /L); Hemoglobin (Hb ≥8.5 g/dL) ; Platelet (PLT ≥ 75 x 10^9/L) Adequate liver function, defined as: Albumin ≥ 2.8 g/dL, Bilirubin ≤3.0 mg/dL, Aspartate aminotransferase (AST), alkaline phosphatase (ALP) and alanine aminotransferase (ALT) were ≤ 5 times the upper limit of normal (ULN) Adequate coagulation function, defined as: International Normalized Ratio (INR) of 2.3 or less Adequate renal function, defined as: creatinine clearance > 40 mL/min, calculated according to the Cockcroft and Gault formula Adequate pancreatic function, defined as: amylase and lipase ≤ 1.5 times ULN
9	Adequate blood pressure (BP) control with up to 3 antihypertensive drugs, defined as: BP ≤150/90 mmHg at screening, and there is no change for antihypertensive therapy within 1 week prior to Cycle 1/Day 1
10	The patient is expected to survive more than 3 months
11	No pregnancy or planned pregnancy
12	Witten informed consent
**Exclusion criteria**	
1	Extrahepatic metastasis
2	Diffuse liver cancer
3	Patients who have received targeted drugs and immune checkpoint inhibitors in the past
4	Hypersensitivity to lenvatinib or PD-1 inhibitor components
5	Patients with myocardial ischemia or myocardial infarction of grade II or above, and poorly controlled arrhythmias (including QTc interval ≥ 470 ms); according to the NYHA standard, grade III to IV cardiac insufficiency, or cardiac color Doppler ultrasonography indicates left ventricular ejection Blood fraction (LVEF) <50%
6	Abnormal coagulation function : INR>1.5 or prothrombin time (PT) > ULN + 4 seconds or activated partial thromboplastin time (APTT) >1.5 ULN, with bleeding tendency or receiving thrombolytic or anticoagulation therapy
7	Pregnant or breastfeeding women; patients with childbearing potential who are unwilling or unable to take effective contraceptive measures
8	Have a history of mental illness or abuse of psychotropic substances
9	Combined HIV-infected
10	History of liver resection, liver transplantation, interventional therapy, and other malignant tumors
11	Patients with active infection
12	With contraindications to radiotherapy
13	Patients with poor compliance such as floating population
14	Those who have participated in clinical trials of other experimental drugs or devices within four weeks
15	Those deemed unsuitable for inclusion by the investigator

#### Interventional methods

Eligible patients will receive radiotherapy for PVTT and primary tumor. CT simulation localization will be performed before radiotherapy and the CT scan images will be transmitted to the treatment planning system in preparation for target delineation. The gross tumor volume will include liver tumor lesions and portal vein tumor thrombus displayed on the CT image. The clinical target volume margin will be 0.5cm for the liver tumor lesions and no expansion for portal vein tumor thrombus lesions. The interfractional margin will be set at 0.5cm and combined with internal motion compensation to form a field-specific planning treatment volume. RT dose is 30Gy (3Gy*10fractions). RT will be given from Monday to Friday, and will be finished in two weeks. Lenvatinib is started on the first day of radiotherapy (daily dose determined according to body weight, 8mg for bodyweight < 60 kg and 12 mg for bodyweight ≥ 60 kg) and will be discontinued 7 days before surgery.

**Figure 2:**
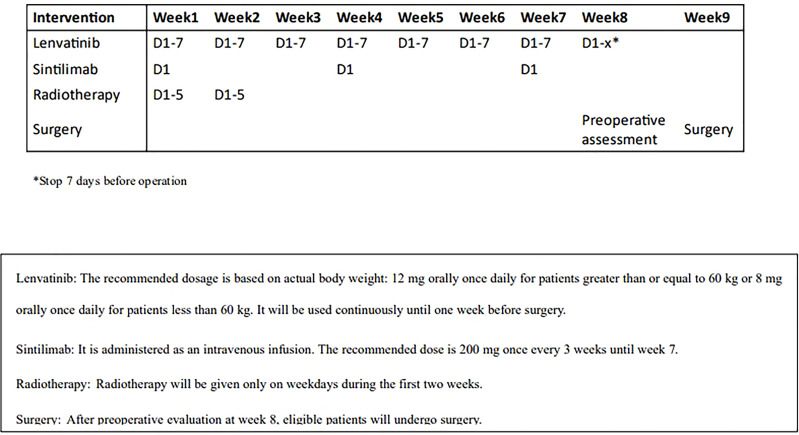
Clinical trial process.

The PD-1 inhibitor(sintilimab) is also started on the first day of radiotherapy, with a fixed dose of 200 mg every three weeks for three cycles. Surgery will be performed 6 to 8 weeks after radiotherapy (**Figure 2**). Dose adjustment, interruption, or discontinuation of lenvatinib and sintilimab according to the adverse events (AES) is detailed in **Tables 2**, **3**.

**Table 2:** Dose adjustment criteria of lenvatinib according to the AEs.

AEs	Degree of AEs	Management	Taper and resume lenvatinib mesylate
Hpertension	Grade 3 (despite optimal antihypertensive therapy)	Suspend	Remission to grade 0, 1 or 2.
	Grade 4	Permanently discontinue	Treatment must not be restarted
Pteinuria	≥ 2 g/24 hours	Suspend	Remission to less than 2g/24 hours
Nephrotic syndrome	-------	Permanently discontinue	Treatment must not be restarted
Renal insufficiency or kidney failure	Grade 3	Suspend	Remission to Grade 0-1 or Baseline
	Grade 4*	Permanently discontinue	Treatment must not be restarted
Heart dysfunction	Grade 3	Suspend	Remission to Grade 0-1 or baseline
	Grade 4	Permanently discontinue	Treatment must not be restarted
Posterior Reversible Encephalopathy Syndrome (PRES) / Reversible Posterior Leukoencephalopathy Syndrome (RPLS)	Any grade	Suspend	If remission reaches grade 0-1, consider restarting treatment at a reduced dose
Liver toxicity	Grade 3	Suspend	Remission to Grade 0-1 or baseline
	Grade 4*	Permanently discontinue	Treatment must not be restarted
Arterial thromboembolism	Any grade	Permanently discontinue	Treatment must not be restarted
Bleeding	Grade 3	Suspend	Remission to Grade 0-1
	Grade 4	Permanently discontinue	Treatment must not be restarted
Gastrointestinal perforation or gastrointestinal fistula	Grade 3	Suspend	Remission to Grade 0-1 or baseline
	Grade 4	Permanently discontinue	Treatment must not be restarted
Parenteral fistula	Grade 4	Permanently discontinue	Treatment must not be restarted
QT interval prolongation	>500 ms	Suspend	Remission to ≤ 480 ms or baseline
diarrhea	Grade 3	Suspend	Remission to Grade 0-1 or baseline
	Grade 4 (although medically managed)	Permanently discontinue	Treatment must not be restarted

* It can be treated according to Grade 3 adverse reactions if it is judged to be non-life-threatening when the adverse reaction is laboratory abnormal Grade 4.

**Table 3:** Adjustment criteria of PD-1 inhibitor according to the AEs.

AEs	Degree of AEs	Management	Taper and resume lenvatinib mesylate
Pneumonia	Grade 2	Suspend	Remission to grade 0-1.
	Grade 3 or 4 or recurrent grade 2	Permanently discontinue	Treatment must not be restarted
Nephritis	Grade 2, creatinine greater than 1.5 times and less than 3 times of ULN	Suspend	Remission to grade 0-1.
	Grade 3 or 4, creatinine greater than 3 times of ULN	Permanently discontinue	Treatment must not be restarted
Colitis	Grade 2 or 3	Suspend	Remission to grade 0-1.
	Grade 4 or recurrent grade 3	Permanently discontinue	Treatment must not be restarted
Endocrine disease	Adrenal insufficiency Symptomatic hypophysitis Type 1 diabetes with hyperglycemia ≥ grade 3 (fasting blood glucose > 250 mg/ml or 13.9 mmol/L) or related ketoacidosis Hyperthyroidism ≥ grade 3	Suspend	Remission to Grade 0-1. For patients with grade 3 or 4 endocrine disease who have improved to grade 2 or lower, and who have clinical symptoms that can be controlled by hormone replacement, consider continuing PD-1 inhibitors after reducing the dose of corticosteroids, otherwise treatment should be discontinued. Hypothyroidism can be managed with replacement therapy without interruption of treatment.
Hepatitis	Grade 2, ALT or AST > 3-5 times ULN or TBIL > 1.5-3 times ULN	Suspend	Remission to Grade 0-1.
	Grade 3-4, ALT or AST > 5 times ULN or TBIL > 3 times ULN	Permanently discontinue	Treatment must not be restarted
Skin reaction	Grade 3 or suspected Stevens-Johnson syndrome (SJS) or toxic epidermal necrolysis (TEN)	Suspend	Remission to Grade 0-1.
	Grade 4 or confirmed SJS or TEN	Permanently discontinue	Treatment must not be restarted
Other immune-related adverse reactions	Depending on the severity and type of reaction, grade 2 or 3	Suspend	Remission to Grade 0-1 or baseline
	Grade 3 or 4 myocarditis Grade 3 or 4 encephalitis Grade 3 or 4 Guillain-Barre syndrome	Permanently discontinue	Treatment must not be restarted
	Grade 4 or recurrent grade 3	Permanently discontinue	Treatment must not be restarted
Infusion-related reactions	Grade 3 or 4	Permanently discontinue	Treatment must not be restarted

### Assessment

#### Tumor response assessment

Baseline CT/MRI scan will be performed within 28 days prior to the first treatment. The second CT/MRI scan will be done before surgery. Baseline and the second assessment must follow the same radiological procedures, which include chest CT and abdomen CT/MRI. RECIST v1.1 is utilized for assessment of treatment response.

#### Safety assessment

Routine blood tests, liver and kidney function tests will be performed once a week during the first two weeks. Laboratory tests such as blood routine, liver and kidney function, troponin-T, serum cortisol, and adrenocorticotropic hormone will be performed (once every three weeks for the rest before operation) to evaluate the safety of the treatment. The dosage will be adjusted according to the instructions if the patients have any safety issues.

#### Follow-up

Within one year after the operation, imaging evaluations will be performed every 3 months which including contrast-enhanced CT/MRI of the upper abdomen and plain CT of the chest. In the second year after surgery, imaging assessments will be performed every 6 months until subjects experience disease progression. After progression, survival follow-up will be performed every 6 months. Due to the small sample size of this study, if there will be a loss of follow-up in the following process, it is necessary to supplement the sample size to ensure 20 enrolled people. Clinical visit information is detailed in **Table 4**.

**Table 4:** Clinical visit information diagram.

	Screening period	Neoadjuvant Therapy(RT+ Sintilimab +Lenvatinib)			Operation			All eligible patients(Non-operated patients will be counted from the day of neoadjuvant failure and surgical patients will be counted from the first postoperative day)		
		Neoadjuvant therapy week 2 (during radiotherapy)	Neoadjuvant therapy week 4(before the start of cycle 2 of sintilimab)	Neoadjuvant therapy week 7(before the start of cycle 3 of sintilimab)	Before Operation	During Operation	1 month ± 10 days after operation	3 month ± 10 days	1st year every 3 months ±10 days	From 2^nd^ year, every 6 months ± 10 days until disease progression or death
Demographics	**√**									
Past medical history	**√**									
Vp typing of PVTT	**√**							**√**		
Child-Pugh score	**√**			**√**	**√**		**√**			
ECOG score	**√**			**√**	**√**		**√**	**√**	**√**	**√**
ICGR-15	**√**				**√**					
Routine blood test	**√**	**√**	**√**	**√**	**√**		**√**	**√**	**√**	**√**
Liver and kidney function test	**√**	**√**	**√**	**√**	**√**		**√**	**√**	**√**	**√**
Thyroid function test	**√**			**√**						
ACTH test (8:00 am.)	**√**			√						
Serum cortisol test (8:00 am.)	**√**			**√**						
Troponin	**√**		**√**	**√**						
AFP、PIVKA-II	**√**				**√**		**√**	**√**	**√**	**√**
immune-related biomarkers(PD-L1、TMB)							**√**			
Immune cell typing (CD4+ T cells, CD8+ T cells)	**√**				**√**		**√**			
Abdominal image	**√**				**√**		**√**	**√**	**√**	**√**
Lung imaging	**√**				**√**			**√**	**√**	**√**
ECG	**√**				**√**					
Inclusion and exclusion criteria judgment	**√**									
Neoadjuvant therapy		**√**	**√**	**√**						
Operation						**√**				
Efficacy evaluation				**√**				**√**	**√**	**√**
Adverse event		**√**	**√**	**√**	**√**	**√**	**√**	**√**	**√**	**√**
Concomitant medication/therapy		**√**	**√**	**√**			**√**	**√**	**√**	**√**

#### Sample size

The main study center treated 5 HCC patients with PVTT (all VP4) between 2020 and 2022. These patients underwent surgery after using the same neoadjuvant therapy as this trial. All patients achieved good efficacy, with a postoperative MPR of 60% (3/5), and manageable safety profile, supporting this trial. Based on this result, we set the end-point of effective response rate as 60%. We simulated response rates of 10%, 20%, and 30% assumed as pathological effective naturatly before intervention. The recruiting time for whole population of the patients is 24 months, and the last enrolled patient will be followed for 12 months. Two-sided Tests with 80% power is used with type I error set at 0.05, and one-Sample tests for exponential hazard rate is used to calculate sample size. The sample size calculate by PASS software 2021 version is 6, 10 and 18 for an effective response rate 10%, 20% and 30%, respectively. Based on the maximum possible effective response rate as control at baseline, we finally set it as 30% and obtained a sample size of 18 that included 11 events. The final sample size is 20 patients, allowing for a 10% loss to follow-up.

#### Statistical analysis

This is an open-label, single-arm, phase I clinical trial and a planned 20 eligible subjects will be enrolled. Descriptive Analysis: The description of the quantitative indicator will give the median. Baseline Demographic Analysis: Descriptive analysis of baseline demographic data, and the chi-square test or survival analysis for numerical data in the experimental and control groups, depending on the data type. Analysis of evaluation indicators: Safety and feasibility, MPR rate, 1-year recurrence-free survival rate, ORR, PVTT regression rate, IPCR, ECOG score, tumor marker changes (AFP, PIVKA-II), ICG-R15 Changes, changes in liver function, etc. analysis of variance using two independent samples t-test, nonparametric test, repeated measures design according to data type. OS and RFS will use survival analysis according to data type. Safety evaluation: Adverse events will be described by the number and incidence, and detailed descriptions of the specific manifestations and degrees of all adverse events and their relationship with the drugs used. Since it is an exploratory study, the research data and results are subject to the investigator’s evaluation, and a Data Monitoring Committee (DMC) is not specially established.

#### Outcome definitions

- Safety: the number of patients who reported incidence of grade ≥3 treatment-related adverse events (according to CTCAE v5.0);
- Feasibility: the number of patients who complete pre-op treatment and proceed to surgery;
- Major Pathological Response (MPR): a reduction in the proportion of surviving tumors below a clinically significant cutoff (≤10% of surviving tumors);
- 1-year recurrence-free survival: the proportion of all patients without HCC recurrence one year after liver resection;
- Objective Response Rate (ORR): the percentage of patients with complete response (CR) and partial response (PR) in all patients, and the response to treatment is based on the modified Response Evaluation Criteria in Solid Tumors (RECIST 1.1);
- Imaging-Pathology Concordance Rate (IPCR): the proportion of all patients with consistent PVTT regression on preoperative imaging and postoperative pathological PVTT regression assessment;
- PVTT regression rate: the proportion of patients with PVTT regression after treatment, divided into PVTT regression rate assessed by imaging and PVTT regression rate assessed by pathology;
- Median overall survival (mOS): the median difference (in months) between the date of study enrollment and the date of death due to any cause. Patients still alive at the end of the study will also be treated as censored, with the last known survival date as the last survival time;
- Recurrence-free survival (RFS): from radical resection to the date of the first documented tumor into recurrence or death from any cause, whichever occurred first.

## Anticipated results

Safety and feasibility will be used as a primary endpoint, and MPR, one-year recurrence-free survival, ORR, IPCR, PVTT regression rate, OS and RFS will be as secondary endpoints. We expect to observe the safety data and the surgical conversion rate of the study group in order to assess the feasibility of subsequent phase II clinical study. And we will use these outcomes to determine the sample size of the further clinical study.

## Discussion

Portal vein tumor thrombus (PVTT) is a common phenomenon in hepatocellular carcinoma (HCC) patients, classified by the VP classification of Japan (15) or Cheng’s type of China in clinical practice. VP classification appeared earlier and was more widely used worldwide so in this study VP classification will be used as the classification standard of PVTT.

Our protocol explores the safety and efficacy of radiotherapy combined with lenvatinib and PD-1 inhibitor (sintilimab) as neoadjuvant therapy for hepatocellular carcinoma complicated with PVTT, which has good prospects.

First, radiotherapy is an effective treatment modality for PVTT. A previous report pointed out that the response rate of radiotherapy in patients with different PVTT classifications was 32.6%-100%, and the 5-year survival rate and the median OS were 5.1%-58.0% and 5.3 to 27.0 months, respectively (16). Low-dose radiotherapy can enhance immunity, which provides a curative effect in PVTT than liver tumors. A Japanese study performed 30-60Gy/10-12F radiotherapy on the tumor thrombus of the main portal vein and its branches, and then surgical resection was performed within 2 weeks after radiotherapy. The results showed that the postoperative PCR rate reached 53%. Another controlled study in China found that neoadjuvant radiotherapy improved PFS and OS in PVTT patients, with the radiation dose of only 3Gy*6F. Low-dose radiotherapy reducing radiation damage to normal tissues and organs can effectively reduce the incidence of adverse reactions, improving the quality of life. In this study, the radiotherapy dose was determined to be 3Gy*10F.

Second, combined targeted and immune therapy is an effective treatment for HCC, having a synergistic effect with radiotherapy. The improvement of the objective response rate of systemic therapy drugs, such as various anti-angiogenic drugs and immune checkpoint inhibitors (ICIs), has brought more possibilities for preoperative treatment. Moreover, low-dose radiation converts TAM to M1 phenotype, infiltrating existing T cells into tumors, thereby promoting the transformation of tumors from “cold” to “hot” (17, 18). In addition to that, some clinical studies suggested that the combination of radiotherapy and immunization may benefit the survival of patients with unresectable HCC (19, 20).

Third, a few studies recently have explored neoadjuvant therapy of HCC. The final results of nivolumab alone or in combination with ipilimumab in the perioperative period of resectable HCC, a phase II randomized controlled, open-label study, were reported at the 2020 ASCO meeting. The results demonstrated that among 27 evaluable patients, the pCR rate was 19%, with 21 patients undergoing planned surgery (21). Another study in mildly resectable or locally advanced HCC reported at ASCO-GI 2021 showed that neoadjuvant therapy of cabozantinib combined with nivolumab achieved margin-negative resection in 12 of 15 patients, of which 5 were major/complete pathological response (22).

To date, there are no study on neoadjuvant therapy using lenvatinib and PD-1 inhibitor combined with radiotherapy for BCLC stage C. This study will provide preliminary evidence for the safety and efficacy of radiotherapy combined with lenvatinib plus PD-1 inhibitors (sintilimab) as neoadjuvant therapy for resectable HCC with PVTT. In addition, some interesting questions, such as the PVTT regression rate with neoadjuvant therapy and the consistency of imaging and pathological assessment of PVTT regression rates, will be explored in this study. In summary, this study may supplement the clinical decision-making evidence in the neoadjuvant treatment of BCLC stage C patients.

## Data availability statement

The original contributions presented in the study are included in the article/supplementary material. Further inquiries can be directed to the corresponding authors.

## Ethics statement

The studies involving human participants were reviewed and approved by ethics committee of Beijing Tsinghua Changgung Hospital. The patients/participants provided their written informed consent to participate in this study.

## Author contributions

JD was the principle investigator of the study who supervises and coordinates the whole project. SY and GoL as principle investigators were involved in the study conception and design. GuL, BS, ZZ, HY, CZ, YX, YY will be involved in the acquisition of data. GoL and GuL will be involved in neo-adjuvant therapy. SY and BS will be involved in surgery. ZZ and CZ will be involved in imaging assessment. HY and YX will be involved in pathological evaluation. YY and ZY will be responsible for follow-up of the enrolled patients. GuL and BS will be involved in the analysis and interpretation of data. GuL were involved in draft in the manuscript. XZ will be in charge of data statistics. GuL and BS were involved in revising the manuscript. All authors contributed to the article and approved the submitted version.

## Funding

This work is supported by CAMS Innovation Fund for Medical Sciences (grant number: 2019-I2M-5-056); National Natural Science Foundation of China (81930119, 82090052, 82090053). The authors declare that they have no competing interests relative to this study.

## Acknowledgments

We thank all the investigators who have contributed to this study.

## Conflict of interest

The authors declare that the research was conducted in the absence of any commercial or financial relationships that could be construed as a potential conflict of interest.

## Publisher’s note

All claims expressed in this article are solely those of the authors and do not necessarily represent those of their affiliated organizations, or those of the publisher, the editors and the reviewers. Any product that may be evaluated in this article, or claim that may be made by its manufacturer, is not guaranteed or endorsed by the publisher.
